# Supplementary material for: Shared latent genetic liability across fibromyalgia and psychiatric traits: Novel insights from genomic structural equation modeling
Source: PLoS Genet. 2026 Jan 23;22(1):e1012034. doi: 10.1371/journal.pgen.1012034 (PMC12867332; doi:10.1371/journal.pgen.1012034)
Supplement: Attachment — Submitted filename: Response to Reviewers.docx [file pgen.1012034.s021.docx]

**Point-to-Point Response**

Reviewer #1: Overview:
The authors aim to investigate genetic underpinnings related to chronic pain and psychiatric disorders by leveraging the genetic overlap among four GWAS summary statistics, including fibromyalgia, insomnia, depression, and anxiety disorder. They utilize the Genomic Structural Equation Modeling method to conduct a common factor analysis with these four traits, resulting in a combined genetic factor (mvFibroPsych). After verifying the fit indices of this model, they conduct thorough follow up analysis (gene, gene-set, tissue enrichment, latent causal variable method, QTL analysis, brain- and proteome-wide based mendelian randomization) to annotate genetic effects and interpret these as underlying the shared etiology among the four disorders.
 The research is comprehensive insofar as it gathers available data from multiple cohorts and combines these in GWAS meta-analyses to increase power for their research design. However, I have fundamental concerns about this research design and how it pertains to the authors’ research questions.
**Response:** *We sincerely thank the reviewer for the thoughtful and constructive feedback. We are especially grateful for the recognition of the comprehensive scope of our work, including the integration of multiple GWAS summary statistics and the use of GenomicSEM to explore the shared genetic architecture across fibromyalgia, insomnia, depression, and anxiety.*

Major comments:
-Across the four disorders, the number of GWAS cases as listed in supplementary table 1 amounts to 216,133 cases. Out of these, fibromyalgia cases amount to 2% (4,528) cases. Any latent factor comprised of these four traits is thus likely to mainly reflect genetic liability for the most powered traits (insomnia, depression, anxiety) and be less relevant for fibromyalgia. This is further exacerbated by the fact that only about half of the genetic variance of fibromyalgia is explained by the common factor model (i.e., the residual variance of fibromyalgia in the common factor model is 46%). Despite of this, the authors frequently interpret the results in the discussion as equally relevant to “chronic pain and psychiatric disorders”. I am hesitant of their interpretation as the result associated with the common factor will reflect the statistical power of the traits they include, of which fibromyalgia is the lowest powered one. Additional sanity checks are needed to verify that the author's interpretation is valid.
1. Investigate the individual contribution of each trait to the reported results for all follow-up analyses by conducting and presenting the same analysis on each of the four traits separately. If the effect of fibromyalgia is not nominally significant for e.g., a result in the QTL analysis then a more likely interpretation is that the effect is completely driven by higher power of depression/insomnia/anxiety and not relevant for fibromyalgia.

**Response:** *We thank the reviewer for the thoughtful and constructive comments. We greatly appreciate the opportunity to address the concern regarding the imbalance in GWAS case numbers across the four traits, particularly the relatively smaller sample size for fibromyalgia compared to insomnia, depression, and anxiety. Below, we address this point in detail and clarify how we have considered statistical power, trait-specific contributions, and the rationale for using GenomicSEM in this context.*

*While we fully acknowledge that sample size and statistical power are important considerations in many genetic analyses, we respectfully argue that the use of GenomicSEM in this setting is both methodologically appropriate and advantageous. One of the core motivations for applying GenomicSEM is precisely to overcome limitations related to power disparities among traits. By modeling the genetic covariance structure across multiple phenotypes, GenomicSEM enhances statistical power and allows for the estimation of latent genetic factors, even when some traits (such as fibromyalgia) have relatively modest sample sizes.*

*This strength has been emphasized in foundational work (Grotzinger et al., Nature Human Behaviour, 2019), and other studies such as Rosoff et al. (Nature Aging, 2023) and Chen et al. (Nature Genetics, 2023). Notably, in the study by Chen et al., cases in individual traits contributed anywhere from 0.216% to 19.9% of the total sample size, yet interpretable and replicable latent factors were uncovered. These findings reinforce that sample size imbalance across traits is not inherently problematic within the GenomicSEM framework, provided there is adequate genetic covariance.*

*As mentioned above, GenomicSEM not only captures shared genetic liability but also increases the effective power for underrepresented phenotypes by leveraging their correlation with more powered traits. This is particularly relevant for downstream analyses such as QTL mapping, etc. In such analysis, it is methodologically expected that findings associated with the latent common factor may not always reach nominal significance (e.g., P < 0.05) when tested in individual phenotypes, especially those with smaller sample sizes. Such discrepancies should not be interpreted as evidence of trait irrelevance, but rather as reflecting the possibility of power limitations in univariate analyses—an interpretation supported by both GenomicSEM theory and multivariate GWAS literature.*

*Finally, we acknowledge that some of our initial language may have overgeneralized the interpretation of our findings as pertaining to “chronic pain and psychiatric disorders” broadly. We appreciate this important feedback and have revised the manuscript accordingly to more precisely state that our findings reflect a shared genetic component between fibromyalgia and psychiatric conditions, rather than chronic pain conditions as a whole. For example, in our prior work, chronic pain phenotypes such as low back pain—with larger sample sizes than fibromyalgia—did not load significantly onto similar latent factors, reinforcing that fibromyalgia may have unique shared etiology with psychiatric traits that is not solely a function of sample size.*

*We hope these clarifications adequately address the reviewer’s concerns and strengthen the overall robustness of our conclusions.*

**References**

- Grotzinger et al. (2019). Genomic structural equation modelling provides insights into the multivariate genetic architecture of complex traits. Nat Hum Behav. 2019 May;3(5):513-525.
- Rosoff et al. (2023). Multivariate genome-wide analysis of aging-related traits identifies novel loci and new drug targets for healthy aging. Nat Aging. 2023 Aug;3(8):1020-1035.
- Chen et al. (2023). Genomic atlas of the plasma metabolome prioritizes metabolites implicated in human diseases. Nat Genet. 2023 Jan;55(1):44-53

2. How does the presented results differ if you only run the common factor model with depression, insomnia and anxiety? If there is little difference between when fibromyalgia is or is not included in the model, then the respective effects are likely not relevant for chronic pain.
-Additional alternative genomic SEM models should be attempted, e.g., fix the factor loading of fibromyalgia to be completely explained by the latent factor. Another idea is to run pairwise models with fibromyalgia and depression / anxiety / insomnia separately to avoid findings being driven by two highly correlated psychiatric disorders.

**Response:** *We thank the reviewer for this thoughtful and methodologically rigorous suggestion. To comprehensively evaluate the contribution of fibromyalgia to the latent genetic factor—and to ensure that the observed effects were not disproportionately driven by the more highly powered psychiatric traits—we conducted a series of sensitivity analyses using alternative GenomicSEM model configurations.*

*Specifically, we implemented the following models: (1) a three-trait common factor model including only depression, anxiety, and insomnia (excluding fibromyalgia); (2) three additional three-trait models, each incorporating fibromyalgia alongside two psychiatric phenotypes (e.g., fibromyalgia + depression + insomnia); and (3) a set of pairwise models in which the factor loading of fibromyalgia was fixed to 1, while the loading of the psychiatric trait was freely estimated.*

*All models demonstrated acceptable fit (CFI > 0.95, SRMR < 0.08). In all three-trait configurations that included fibromyalgia, the fibromyalgia phenotype exhibited statistically significant factor loadings, ranging from 0.70 to 0.81. In the pairwise models, where factor loading of fibromyalgia was fixed to 1, the psychiatric traits also showed significant loadings onto the latent factor—0.71 for anxiety, 0.77 for depression, and 0.76 for insomnia—indicating robust shared genetic architecture across models.*

*These findings demonstrate that fibromyalgia contributes non-redundant genetic variance to the latent construct. Importantly, this contribution is observed despite the relatively modest sample size of fibromyalgia, highlighting the utility of GenomicSEM in leveraging cross-trait genetic covariance to enhance power for underpowered phenotypes.*

*These sensitivity analyses have been summarized in Table S3, and a dedicated description has been added to the revised Methods and Results sections. Together, we provide convergent evidence supporting the meaningful role of fibromyalgia in the shared latent factor, beyond being a statistical byproduct of larger, better-powered psychiatric traits.*

-In UK Biobank there additional cases of fibromyalgia present. UKB was used in meta-analyses of insomnia/depression/anxiety, why not extend the fibromyalgia GWAS meta-analysis with UKB sample? For question Ever had Fibromyalgia syndrome (data field 120009) there are 3,010 additional cases. Alternatively, going by health records, there are 4,891 UKB participants diagnosed with Fibromyalgia (ICD10 code M79.7).
**Response:** *We thank the reviewer for this insightful suggestion. We fully agree that additional fibromyalgia cases in the UK Biobank (via self-report or ICD10 codes) may be valuable for future GWAS meta-analyses.*

*However, our present study was based on publicly available and fully processed GWAS summary statistics, and we did not have access to UK Biobank individual-level genetic and phenotypic data for fibromyalgia. At the time of this study, there were no validated or published GWAS summary statistics for fibromyalgia derived from UKB that could be incorporated directly into our meta-analysis.*

*We agree that incorporating UKB-derived fibromyalgia GWAS in future studies could help strengthen power and improve cross-cohort harmonization, and we plan to pursue this direction as data access and resources allow. We have added a statement in the Methods to clarify this limitation.*

Minor comments:
-In Figure 2A and in-text: I miss standard errors and p-values of the genetic correlations.
-Figure 2C: novel factor hits; what are these 'novel' in relation to? Have none of these hits been associated with any of the included traits? What is the definition of indicator hits?
-Page 20, line 416-418: I find this claim circular. Is not the positive association between the mvFibroPsych and mood disorders expected as depression feature heavily in the latent model?

**Response:** *We thank the reviewer for these helpful and detailed observations. We have addressed each point below:*

*We appreciate the request for more complete statistical reporting. In the revised version, we have now included standard errors and p-values for all pairwise genetic correlations. These values are also reported in the main text and in Table S2 for clarity and reproducibility.*

*We thank the reviewer for requesting clarification regarding the terminology used in Figure 2C. In the revised version, we have now explicitly defined the terms “novel hits” and “indicator hits” to improve clarity. Specifically, “novel hits” are now referred to as “novel lead SNPs”, denoting genome-wide significant loci (P < 5×10⁻⁸) identified in the mvFibroPsych GWAS that have not been previously reported as significant in any of the eight source GWAS datasets (fibromyalgia, insomnia, depression, anxiety). Likewise, “indicator hits” have been renamed as “lead SNPs”, referring to the lead variants identified through FUMA in the mvFibroPsych GWAS. These definitions have been clarified in the Methods section and in the legend of Figure 2C to enhance transparency and interpretability.*

*We agree with the reviewer that the positive genetic correlation between mvFibroPsych and mood disorders is, to some extent, expected given that depression and anxiety contribute substantially to the latent factor. Our original intent was to empirically confirm the external validity of mvFibroPsych by showing that it relates meaningfully to independent GWASs of mood disorders beyond the modeling set. However, we recognize that the current wording may appear tautological. We have revised the sentence to emphasize this nuance more clearly:*

“The observed positive genetic correlation between mvFibroPsych and mood disorders is consistent with the structure of the latent factor, and supports the construct validity of mvFibroPsych by linking it to broader mood-related phenotypes in independent datasets.”

*We appreciate the reviewer for the insightful comments and have revised both the figures and main text.*

Reviewer #2: This study explores the genetic and neurobiological architecture of fibromyalgia, insomnia, depression, and anxiety, using GenomicSEM to identify a latent common factor (mvFibroPsych) and conducting GWAS, Mendelian Randomization (MR), and LCV analyses to assess causal relationships. The authors integrate data from UK Biobank, FinnGen, iPSYCH, and BioMe Biobank to strengthen their findings. Their brain-wide MR analysis highlights a negative causal association between fractional anisotropy in the splenium of the corpus callosum and mvFibroPsych, suggesting white matter integrity in this region may have a protective effect against these comorbid conditions. This underscores the neurobiological component of shared genetic risk. While the study is methodologically rigorous and addresses an important research question, a few aspects require further clarification to ensure robustness and interpretability. Below, I outline key concerns and recommendations to improve clarity and validity.
**Response:** *We sincerely thank the reviewer for the careful review and valuable comments on our study. We are delighted that you acknowledge the methodological rigor and the importance of the research question. We have carefully considered your concerns and have made the necessary clarifications and improvements to enhance the robustness and interpretability of our study. Below, we address each of your concerns in detail.*

1- The rationale for selecting the common pathway model over alternative pathway models is not clearly justified. Since various models could explain the genetic architecture of these phenotypes, the authors should provide a stronger empirical or literature-based justification for their choice. I suggest two possible approaches to strengthen this aspect: Empirical Model Comparison: The authors could compare multiple twin models (ACE, Cholesky decomposition, independent pathway, and common pathway) to determine which best explains the association between the phenotypes. This would provide a data-driven rationale for selecting the common pathway model. Supporting Literature: If prior twin analyses support the use of the common pathway model for these traits, referencing relevant studies in the introduction and methods would help justify this choice. Clarifying this would enhance methodological transparency and reinforce the robustness of the model selection.
**Response:** *We thank the reviewer for this thoughtful and methodologically rigorous suggestion. We fully agree that articulating the rationale for selecting the common pathway model is crucial for methodological transparency and robustness of inference.*

*As the reviewer noted, various alternative models—such as independent pathway or Cholesky decomposition—could in theory be informative for understanding the shared genetic architecture of these phenotypes. However, due to the nature of our data (i.e., GWAS summary statistics) and the current limitations of the GenomicSEM framework, which is specifically designed for use with summary-level data, we were not able to implement formal model comparisons among these traditional structural equation models).*

*Nonetheless, our modeling approach was guided by both theoretical reasoning and empirical precedent. Specifically, a number of studies have reported moderate to high genetic correlations between fibromyalgia, insomnia, depression, and anxiety (e.g., Jansen et al., Nat Genet, 2019; Purves et al., Biol Psychiatry, 2020; Chang et al., J Pain Res, 2024), suggesting the presence of a shared genetic liability. These findings conceptually support the use of a common latent factor model to capture the underlying genetic overlap.*

*Moreover, our approach aligns with prior GenomicSEM studies (e.g., Grotzinger et al., Nat Hum Behav, 2019; Rosoff et al., Nat Aging, 2023), which have adopted common factor models for genetically correlated traits without requiring exhaustive comparisons with traditional twin-based models. Our model also demonstrated excellent empirical fit (e.g., CFI = 0.999, SRMR = 0.015), supporting its appropriateness in this context.*

*We have now clarified this rationale in the revised Methods section as follows:*

“We selected the common factor model based on prior evidence of substantial genetic correlations among the included traits, and in line with prior GenomicSEM studies that have modeled shared latent genetic liabilities.”

*In addition, we have enhanced the Introduction to provide more background on the genetic and phenotypic relationships among fibromyalgia, insomnia, depression, and anxiety, to further contextualize our modeling approach.*

**References:**

- Jansen et al. (2019) Genome-wide analysis of insomnia in 1,331,010 individuals identifies new risk loci and functional pathways. Nat Genet. 2019 Mar;51(3):394-403.
- Purves et al. (2020) A major role for common genetic variation in anxiety disorders. Mol Psychiatry. 2020 Dec;25(12):3292-3303.
- Chang et al. (2024) Effects of Mental Disorders on Fibromyalgia Mediated by Insomnia: A Mendelian Randomization Study. J Pain Res. 2024 Dec 14:17:4277-4288
- Grotzinger et al. (2019). Genomic structural equation modelling provides insights into the multivariate genetic architecture of complex traits. Nat Hum Behav. 2019 May;3(5):513-525.
- Rosoff et al. (2023) Multivariate genome-wide analysis of aging-related traits identifies novel loci and new drug targets for healthy aging. Nat Aging. 2023 Aug;3(8):1020-1035.

2- The authors used UK Biobank, iPSYCH, and FinnGen for insomnia, depression, and anxiety GWAS summary statistics. However, larger meta-GWAS datasets exist for these traits, such as ISGC (insomnia), PGC-MDD (depression), and PGC (anxiety). Could the authors clarify why these were not used? If unavailable, discussing this limitation and its potential impact on the findings would improve transparency.
**Response:** *We thank the reviewer for this thoughtful and important comment. We fully agree that large-scale meta-GWAS efforts such as ISGC and PGC provide valuable resources and can improve power and generalizability. Below we clarify the rationale for our selection of datasets in this study.*

*For all included phenotypes, we prioritized GWAS summary statistics that were: (1) fully publicly available with genome-wide SNP-level data; (2) free of major sample overlap within each phenotype; and (3) harmonized in terms of ancestry (European), imputation quality, and phenotype definition.*

*Although ISGC and PGC have conducted large meta-GWAS analyses, in many cases, the full summary statistics—including all SNPs and association metrics—are not publicly available, or are restricted due to inclusion of 23andMe or other proprietary cohorts. For example, the Jansen et al. (2019) ISGC insomnia GWAS included ~1.3 million individuals, but the publicly available data exclude 23andMe samples, significantly reducing sample size and variant coverage.*

*Moreover, substantial portions of the ISGC and PGC datasets overlap with UK* *Biobank, iPSYCH, or FinnGen, which were already used in our current analyses. Incorporating such overlapping data without careful modeling of shared individuals could introduce biased genetic covariance estimates, compromising the validity of GenomicSEM results.*

*To balance statistical power with methodological rigor, we therefore selected GWAS summary statistics from UK Biobank, FinnGen, BioMe, and iPSYCH, which provide full summary data and minimize sample overlap across phenotypes.*

*We acknowledge this choice entails a trade-off in statistical power, and have now added a sentence in the Discussion to explicitly state this limitation. In future work, we intend to explore incorporating filtered versions of ISGC/PGC datasets where available and consider overlap correction methods to enable more comprehensive meta-analytic modeling.*

**Reference:**

- Jansen et al. (2019). Genome-wide analysis of insomnia in 1,331,010 individuals identifies new risk loci and functional pathways. Nat Genet. 2019 Mar;51(3):394-403

3- The authors conducted a meta-analysis combining UK Biobank and FinnGen data instead of using FinnGen as an independent replication cohort. This raises concerns about potential sample-specific biases and the ability to test the robustness of findings in an external dataset. Could the authors clarify why FinnGen was included in the meta-analysis rather than being used for replication? If independent replication was not feasible, discussing this limitation and its implications would improve transparency.
**Response:** *We thank the reviewer for this insightful comment. We fully acknowledge the importance of independent replication in genetic studies and appreciate the opportunity to clarify our rationale.*

*Our decision to include both UK Biobank and FinnGen in the discovery meta-analysis was driven by the need to maximize statistical power, particularly given the modest number of fibromyalgia cases and the multivariate nature of the GenomicSEM framework. Constructing a stable latent factor (mvFibroPsych) and performing robust downstream analyses—such as tissue enrichment and Mendelian randomization—required well-powered GWAS summary statistics with sufficient SNP density and coverage.*

*Furthermore, GenomicSEM requires accurate estimation of cross-trait genetic covariance matrices, which are more stable when based on larger sample sizes. Using UK Biobank alone would have substantially limited power and the reliability of the multivariate model.*

*We agree that this strategy limits our ability to assess replication in an entirely independent cohort. At the time of analysis, we did not have access to a suitable external dataset that met all necessary criteria (i.e., availability of GWAS for all four phenotypes, European ancestry, consistent QC, and no major sample overlap).*

*We have now explicitly acknowledged this limitation in the revised Discussion section. Once again, we thank the reviewer for this important suggestion, which has strengthened the transparency of our study design.*

4- The genomic control factor (λGC = 1.207) and LDSC intercept (0.835, SE = 0.013) indicate a discrepancy—inflated test statistics despite a deflated intercept. While some inflation is expected in polygenic traits, this pattern may suggest heterogeneity across datasets rather than true polygenicity. Could the authors report the attenuation ratio (LDSC Intercept - 1 / λGC - 1) to assess whether inflation is driven by true polygenicity or residual confounding? If the ratio is high, it would support polygenic architecture as the primary driver of inflation.
**Response:** *We thank the reviewer for this thoughtful and technically important comment. We fully agree that interpreting inflation metrics such as λ_GC_ and LDSC intercept requires care, particularly in multivariate analyses.*

*In our study, we observed a genomic control factor (λ_GC_) of 1.207 and an LDSC intercept of 0.835 (SE = 0.013). While this combination may appear inconsistent at first glance, it has been reported in several GenomicSEM-based multivariate GWAS and is generally interpreted as resulting from a combination of sparse polygenic signal, phenotype heterogeneity across cohorts, and conservative correction of test statistics (e.g., de la Fuente et al., 2021; Wendt et al. 2021; Morey et al. 2024).*

*To further evaluate the nature of this inflation, we calculated the attenuation ratio:*

$$Attenuation Ratio=\frac{LDSC Intercept - 1}{\lambda GC - 1}=\frac{0.835-1}{1.207-1}\approx-0.797$$

*This negative attenuation ratio is atypical in single-cohort GWAS, but has been observed* *in multivariate settings, particularly when traits are genetically sparse or when stringent correction procedures (e.g., inclusion of principal components or mixed model residualization) lead to deflation of the intercept. Importantly, we also verified that LDSC heritability Z-scores and cross-trait intercepts showed no evidence of uncontrolled population structure.*

*We have now reported the attenuation ratio in the Results section and added discussion with references to comparable findings in the Discussion section. We acknowledge this as a potential indicator of trait-specific heterogeneity or overcorrection and will address this more directly in future studies with harmonized phenotyping. We thank the reviewer again for prompting this important clarification.*

**References:**

- Fuente et al. (2021) A general dimension of genetic sharing across diverse cognitive traits inferred from molecular data. Nat Hum Behav. 2021 Jan;5(1):49-58
- Wendt et al. (2021) Multivariate genome-wide analysis of education, socioeconomic status and brain phenome. Nat Hum Behav. 2021 Apr;5(4):482-496
- Morey et al. (2024) Genomic structural equation modeling reveals latent phenotypes in the human cortex with distinct genetic architecture. Transl Psychiatry. 2024 Oct 24;14(1):451

5-The authors applied both Mendelian Randomization and Latent Causal Variable analysis but did not clarify their rationale for selecting one method over the other for specific exposures. Since MR estimates direct causal effects while LCV assesses genetic causality via genetic correlations, could the authors specify their criteria for choosing between these approaches? Was the availability of strong instrumental variables a determining factor? A brief justification in the methods section would improve clarity and transparency.
**Response:** *We thank the reviewer for this insightful comment regarding the rationale behind applying both Mendelian Randomization (MR) and Latent Causal Variable (LCV) analyses. We agree that clarifying the analytic purpose of each method improves transparency, and we have revised the Methods section accordingly.*

*Specifically, we employed LCV to assess the presence and directionality of genetic causality between mvFibroPsych and complex disease traits or modifiable risk factors. LCV estimates the genetic causality proportion (GCP) using genome-wide data and does not require the identification of strong instrumental variables. Thus, it is particularly suited to screening for potential causal architecture when the goal is to detect signal direction rather than quantify effect sizes.*

*In contrast, we applied MR to examine associations between mvFibroPsych and continuous brain imaging-derived phenotypes, where quantifying the magnitude and direction of effect in interpretable units (e.g., beta coefficients) was a central aim.*

*This distinction—directional inference via LCV versus effect size estimation via MR—has now been explicitly stated in the Methods section to clarify our analytic strategy. We thank the reviewer again for helping improve the methodological clarity of the manuscript.*

6-The reported CFI = 0.999 suggests an almost perfect model fit, which may indicate overfitting or an imbalanced contribution of traits to the common factor. Given that depression has the largest sample size and highest factor loading, could the authors clarify whether this might be driving the model’s fit? Additionally, have they assessed whether sample size disparities across traits (e.g., depression vs. fibromyalgia) could be influencing the covariance structure? A sensitivity analysis adjusting for sample size effects would help ensure that the model is not overly weighted by depression
**Response:** *We thank the reviewer for this insightful comment regarding model fit and the potential influence of sample size disparity across traits.*

*From a methodological perspective, GenomicSEM does not directly weight input traits by their sample size. Rather, it models the genetic covariance structure using LD score regression–derived summary statistics and factor loadings are estimated based on the shared genetic architecture among traits, not based on univariate GWAS power (Grotzinger et al., 2019). Therefore, traits with large sample sizes (e.g., depression) do not automatically dominate the model unless they share disproportionately greater genetic covariance with the others.*

*To empirically address this concern, we conducted a series of sensitivity analyses, including: (i) a three-trait model excluding depression; (ii) three-trait models including fibromyalgia paired with other psychiatric traits; and (iii) pairwise user-defined models in which fibromyalgia’s factor loading was fixed to 1 and the loading of psychiatric traits was freely estimated. Across all configurations, we observed consistent model fit (CFI > 0.95, SRMR < 0.08), and both fibromyalgia and the psychiatric traits loaded significantly onto the latent factor (Table S3). Moreover, exclusion of depression did not lead to collapse of the model or major degradation in fit indices.*

*These findings suggest that the model is not being disproportionately driven by depression, and that fibromyalgia contributes non-redundant variance to the shared genetic component, despite its smaller effective sample size. We have summarized these results in Table S3 and included a clarifying paragraph in the revised Results section.*

**Reference:**

- Grotzinger, A. D., et al. (2019). Genomic structural equation modelling provides insights into the multivariate genetic architecture of complex traits. Nature Human Behaviour, 3(5), 513–525.

7- The authors report significant SNPs from their common factor GWAS but do not mention whether they assessed heterogeneity in SNP effects across the component traits. Given that some SNPs may exhibit trait-specific effects rather than acting through the shared factor, did the authors perform any heterogeneity tests, such as Cochran’s Q statistic or variance partitioning across phenotypes? If not, a brief discussion of potential heterogeneity would improve transparency.
**Response:** *We thank the reviewer for this important point. To assess whether genome-wide significant SNPs from the common factor GWAS exert their effects uniformly across traits or exhibit trait-specific heterogeneity, we performed heterogeneity testing using the Cochran’s Q statistic, as implemented in the GenomicSEM framework.*

*We now report these results in the revised manuscript. Specifically, we identified a subset of significant SNPs with evidence of heterogeneity across component traits (Q_P-value < Bonferroni threshold). This indicates that while many loci likely reflect shared genetic effects captured by the latent factor, a portion of loci may exert stronger or exclusive effects on specific traits. We have now included these findings in the Results section, and a summary of Q statistics and corresponding p-values for genome-wide significant SNPs is provided in Table S5. A brief discussion has also been added to the Discussion section to clarify this important nuance.*

8-Many of the genome-wide significant SNPs have extremely small p-values, which may be influenced by differences in GWAS sample sizes across traits. Since depression GWAS has the largest sample size, could the authors clarify whether depression-associated SNPs disproportionately contribute to the common factor model loadings?

**Response:** *We thank the reviewer for this important point. To address the concern that depression—a trait with the largest sample size—may disproportionately influence SNP associations with the common factor, we examined the Z statistics of all genome-wide significant lead SNPs across the four input phenotypes: fibromyalgia, depression, anxiety, and insomnia.*

*Our results show that many mvFibroPsych-associated SNPs do not have their strongest signal in depression. Several SNPs exhibit higher absolute Z-values in insomnia or anxiety, and a notable proportion even show higher signals in fibromyalgia despite its smaller sample size. This pattern suggests that the top SNPs are not uniformly driven by depression, and instead reflect joint contributions from multiple phenotypes.*

*These findings highlight a core strength of the GenomicSEM framework—its ability to extract shared genetic architecture beyond the dominant influence of a single trait. We have now reported the Z-scores across all traits for lead SNPs in Table S5, and have added a clarifying statement to the Results section.*

Reviewer #3: This article explores the shared genetics and molecular biology between fibromyalgia, anxiety, depression, and insomnia. Fibromyalgia is an idiopathic condition characterized by widespread musculoskeletal pain, and is often comorbid with psychiatric conditions. This begs the question of whether there are shared genetic risks and biological pathways between these conditions. The authors use genomic structural equation modeling (gSEM) and genome-wide association studies (GWAS) to identify associated genetic variants that are shared between these phenotypes, and employ a suite of functional genomics tools to investigate the biology underlying these variants; including FUMA, Latent Causal Variable (LCV) Analysis, brain-wide and proteome-wide Mendelian Randomization (MR) and QTL analysis. I have little domain knowledge of fibromyalgia but have expertise in psychiatric genetics, gSEM, GWAS, and all the downstream analyses except LCV and MR. Overall, the article is very well-written and has excellent flow. The authors also do a great job of explaining the purpose of each analysis and how to interpret the results from each one. My major comments center around including more information about the GWAS analyses.
**Response:** *We sincerely thank the Reviewer for the thoughtful and encouraging comments. We greatly appreciate the acknowledgment of the structure, clarity, and methodological transparency of our work, particularly in areas such as genomic SEM, GWAS, and downstream functional annotation.*

*We are especially grateful for the recognition of our efforts to clearly communicate the purpose and interpretation of each analysis and we are pleased to know that our exposition was helpful.*

ABSTRACT AND INTRODUCTION
Major issues:
Minor issues:
-Clarify that the meta-analyses were within phenotypes, not across phenotypes (see comment in minor issue for methods)
**Response**: *We thank the reviewer for these constructive suggestions. We agree that the original wording in the abstract—“We conducted a GWAS meta-analysis encompassing fibromyalgia, insomnia, depression, and anxiety…”—could be misinterpreted as referring to a cross-phenotype meta-analysis.*

*We have revised the abstract to more accurately reflect this workflow. The sentence now reads as follows:*

“We conducted phenotype-specific GWAS meta-analyses for fibromyalgia, insomnia, depression, and anxiety to enhance statistical power prior to multivariate modeling.”

-Provide more detail about the GWAS including ancestry, and effective sample size.
**Response:** *All GWAS summary statistics used in our analyses were derived from individuals of European ancestry to ensure genetic homogeneity and reduce potential bias in LD Score Regression and GenomicSEM modeling. This information including ancestry, and effective sample size has now been added to the Methods section and summarized in Table S1.*

-Were the 64 novel loci novel for fibromyalgia, the psych conditions, or both?
**Response:** *We thank the reviewer for this helpful clarification. In our study, “novel loci” refer to genome-wide significant lead SNPs (P < 5×10⁻⁸) identified in the mvFibroPsych GWAS that were not reported as significant in any of the eight univariate GWAS datasets used as GenomicSEM input, including those for fibromyalgia, insomnia, depression, and anxiety (with up to two datasets per trait when available).*

*This definition follows the approach used in prior GenomicSEM studies (Rosoff et al., 2023). We have clarified this terminology in the revised Results section and Figure 2 legend.*

**Reference:**

- Rosoff et al., (2023) Multivariate genome-wide analysis of aging-related traits identifies novel loci and new drug targets for healthy aging. Nat Aging. 2023 Aug;3(8):1020-1035.

-In the introduction, please include a paragraph that summarizes previous genetic studies of fibromyalgia, including any recent GWAS.
**Response:** *We thank the reviewer for this invaluable comment. Following by the suggestion, we have added a dedicated paragraph in the revised Introduction, summarizing prior genetic studies of fibromyalgia that reads as:*

“While the psychiatric comorbidities of fibromyalgia are well-documented, its genetic basis remains relatively underexplored. Early candidate gene studies yielded inconsistent findings, and only a few genome-wide association studies have been conducted to date. For example, studies by Docampo et al. and Peters et al. reported suggestive but non-significant associations in small cohorts, while Biobank-based analysis revealed polygenic overlap with psychiatric traits but no genome-wide significant loci (Docampo et al., 2014; Peters et al., 2013; Moscati et al., 2023). These findings point to a modest SNP-based heritability and polygenic architecture for fibromyalgia, underscoring the need for multivariate approaches to uncover shared genetic mechanisms with its common psychiatric comorbidities.”

**References:**

- Docampo et al. (2014) Genome-wide analysis of single nucleotide polymorphisms and copy number variants in fibromyalgia suggest a role for the central nervous system. PAIN®. 2014;155(6):1102-9.
- Peters et al. (2013) Genome-wide association study meta-analysis of chronic widespread pain: evidence for involvement of the 5p15. 2 region. Annals of the rheumatic diseases. 2013;72(3):427-36.
- Moscati et al. (2023) Life is pain: Fibromyalgia as a nexus of multiple liability distributions. American Journal of Medical Genetics Part B: Neuropsychiatric Genetics. 2023;192(7-8):171-82.

METHODS
Major issues:
-There is no information on how the GWAS of mvFibroPsych was run. Please add a paragraph for this topic and include information such as the effective sample size, what software was used to run the analysis, what covariates were used, whether a logistic or linear regression was used, etc.

**Response:** *We thank the reviewer for this helpful comment and agree that greater methodological transparency is essential. In response, we have now added a dedicated paragraph to the Methods section detailing how the mvFibroPsych GWAS was conducted.*

*Specifically, we clarify that the multivariate GWAS was conducted using the commonfactorGWAS() function in the GenomicSEM R package (v* *0.0.5), based on genome-wide summary statistics from four input traits. The model was estimated using diagonally weighted least squares (DWLS). SNPs were filtered using standard QC thresholds, and summary statistics were pre-adjusted for age, sex, principal components, and study-specific covariates. The effective sample size for the mvFibroPsych GWAS was also reported. This detailed information has been added to the Methods section of the revised manuscript.*

Minor issues:
-It is my understanding that you did a meta-analysis of two studies for each phenotype, then fed these four sets of sumstats into LDSC and gSEM. This is not what you describe; rather, you describe doing a meta-analysis ACROSS all phenotypes to generate a single set of sumstats, which was then fed into LDSC and gSEM, which is not possible. Please clarify this point.

**Response:** *We thank the reviewer for this helpful observation and the opportunity to clarify this important methodological point. We confirm that we did not perform a meta-analysis across phenotypes. Rather, as the reviewer correctly inferred, we conducted meta-analyses within each phenotype, combining summary statistics across multiple cohorts, to obtain a final set of GWAS summary statistics per phenotype. These four trait-specific summary statistics—for fibromyalgia, insomnia, depression, and anxiety—were then independently processed and used as input into LDSC to estimate the genetic and sampling covariance matrices. These matrices served as the foundation for the GenomicSEM common factor model, which was subsequently used to estimate the mvFibroPsych factor and perform the multivariate GWAS.*

*We regret any ambiguity in our original description, and we have now revised the Methods section to clarify that the LDSC and GenomicSEM analyses were based on trait-level summary statistics, not on any pooled GWAS across phenotypes. We have now revised the sentence as follows:*

“We conducted *phenotype-specific GWAS me*ta-analyses for fibromyalgia, insomnia, depression, and anxiety, respectively.”

-I know you include the sample sizes for the individual GWAS in the supplement, but please include the effective N for each meta analysis results, and the gSEM GWAS result, in the Methods. The reader will want to know.

**Response:** *We thank the reviewer for this helpful suggestion. In response, we have now explicitly added the effective sample sizes (Neff) for each of the four meta-analyzed input traits (fibromyalgia, insomnia, depression, and anxiety) in the revised Methods section. These were calculated using the standard case–control approximation (Bulik-Sullivan et al., 2019):*

$$Neff=\frac{4\cdot Ncase\cdot Ncontrol}{Ncase+Ncontrol}$$

*This approximation accounts for the reduced statistical information in unbalanced case–control designs and is widely used in LD Score Regression (LDSC) analyses. For meta-analyses of multiple cohorts, we computed Neff for each component dataset separately and summed them to generate the total effective sample size per phenotype. Information of Neff is now provided in Table S1.*

*In addition, we now report the effective sample size for the mvFibroPsych GWAS, which was estimated using the method recommended in the GenomicSEM package. This approach infers multivariate Neff based on genome-wide SNP-wise variances and is widely used in latent factor GWAS.*

**Reference:**

- Bulik-Sullivan et al., (2019) LD Score regression distinguishes confounding from polygenicity in genome-wide association studies. Nat Genet. 2015 Mar;47(3):291-5.

-In the GWAS Meta-analysis section, please include a description of how the phenotypes were defined in the GWAS. Were they binary (case-control) phenotypes? How were cases defined, given that you mention earlier that fibromyalgia is heterogenous?
**Response:** *We thank the reviewer for this helpful comment. In response, we have added a dedicated paragraph to the Methods section to clarify phenotype definitions. All four phenotypes (fibromyalgia, insomnia, depression, and anxiety) were analyzed as binary case–control traits. For fibromyalgia, cases were defined based on the ICD-10 diagnosis code M79.7. Definitions for insomnia, depression, and anxiety followed the criteria used in the original GWAS sources and are now briefly summarized and properly cited in the revised Methods. Full sample details are also provided in Table S1.*

-For the genome-wide association study annotation section, please mention what % of variants were assigned by FUMA to their effector gene based on positional mapping vs which had functional evidence (QTL, chromatin data) supporting their gene assignment
**Response:** *We thank the reviewer for this helpful suggestion. In the revised Methods, we now report the proportions of genome-wide significant SNPs mapped to genes via different strategies, based on FUMA output, where all SNPs in linkage disequilibrium (LD; r² ≥ 0.6) with independent significant SNPs were included. Among these SNPs, 56.7% were mapped via positional mapping, 70.7% via eQTL mapping, and 0% via chromatin interaction mapping, under default FUMA parameters. Some SNPs were mapped through more than one strategy. These results have been added to the Results section and Table S6.*

RESULTS
Major issues:
-At the beginning of the results section, please include a paragraph that summarizes the results of the four GWAS meta-analyses you did. What were the resulting sample sizes for each of the four phenotypes? Did performing the meta-analyses increase the number of significantly associated loci? You mention that you did the meta-analyses to improve power, but don’t say anything about whether they actually did improve power.
**Response:** *We thank the reviewer for this important comment. In response, we have now added a summary paragraph at the beginning of the Results section to describe the outcomes of the four phenotype-specific GWAS meta-analyses. This includes reporting the effective sample sizes (Neff) for each trait after meta-analysis, and a comparison of genome-wide significant loci before and after meta-analysis where applicable. After meta-analysis, the estimated effective sample sizes (Neff) were 17,827 for fibromyalgia, 115,173 for insomnia, 391,264 for depression, and 191,496 for anxiety disorder. In particular, meta-analysis modestly increased the number of genome-wide significant loci, supporting improved statistical power. These improvements are consistent with expectations for polygenic traits where increased sample size improves discovery power. This summary and interpretation are now included at the beginning of the Results section and Table S1.*

Minor issues:
-Typically when a GWAS is run on a gSEM model, there is one paragraph about the gSEM model results and one paragraph about the GWAS results. I would recommend following this format by ending the first paragraph with the sentence ‘the standard factor loadings...” and beginning a new paragraph with the next sentence ‘The common factor model built in the gSEM model’. I would move the first sentence of the next paragraph ‘A total of 122 lead SNPs’ into this new paragraph as well.
**Response**: *We thank the reviewer for this helpful editorial suggestion. In response, we have revised the relevant section of the Results to improve clarity and follow standard reporting structure for GenomicSEM studies as suggested.*

-In the latent common factor GWAS estimation paragraph, please comment on whether the observed genetic correlations are reasonable/expected based on previous studies
**Response**: *We thank the reviewer for this thoughtful suggestion. In response, we have now added commentary in the Results section to contextualize the observed genetic correlations among the four input traits (fibromyalgia, insomnia, depression, and anxiety).*

-Please clarify what you mean by novel when referring to the 64 novel loci. Were they novel associations for fibromyalgia, the psych disorders, or all?
**Response**: *We appreciate the request of the reviewer for clarification regarding the use of the term "novel" in reference to the 64 loci identified in the mvFibroPsych GWAS.*

*In this study, we define “novel” loci as those that did not reach genome-wide significance (P < 5 × 10⁻⁸) in any of the individual GWAS used to construct the GenomicSEM model—namely, fibromyalgia, insomnia, depression, and anxiety disorder. We have clarified this definition in the Results section of the revised manuscript.*

-The figures are blurry, please provide clearer images
**Response**: *We thank the reviewer for bringing this to our attention. In the revised submission, we have replaced figures with high-resolution versions to ensure visual clarity and readability. We appreciate the valuable feedback of the reviewer on improving the presentation of the figures.*

-In Figure 2c, I would suggest using the terms ‘lead SNPs’ and ‘novel lead SNPs’ instead of ‘indicator hits’
**Response:** *We thank the reviewer for this helpful suggestion. In accordance with the recommendation, we have updated the terminology in Figure 2C and throughout the manuscript. Specifically, we now refer to “indicator hits” as “lead SNPs”, and “novel hits” as “novel lead SNPs”, to ensure consistency with standard GWAS nomenclature. The figure legend and related text have been revised accordingly.*

-The description of the LVC results in the result section is a bit confusing; it seems like both descriptions are describing mvFibroPsych causally influencing something:

--‘Among these, 58 phenotypes were found to be causally influenced by mvFibroPsych, whereas mvFibroPsych was identified as a causal factor for 13 phenotypes’
**Response:** *We thank the reviewer for highlighting this ambiguity in our phrasing. We acknowledge that the original sentence may have incorrectly suggested that mvFibroPsych was the causal exposure in both cases, which is not the correct interpretation of the LCV results. To clarify, in 58 traits, mvFibroPsych was identified as the outcome, i.e., these traits may exert a causal genetic influence on mvFibroPsych (negative GCP); while in 13 traits, mvFibroPsych was identified as the exposure, i.e., it may causally influence those traits (positive GCP). We have revised the sentence in the Results section accordingly to make the directionality explicit. The revised sentence now reads as follows:*

“Among these, 58 phenotypes showed evidence of genetically causal effects on mvFibroPsych (negative GCP), while mvFibroPsych itself was identified as a potential causal factor for 13 phenotypes (positive GCP).”

*We thank the reviewer again for pointing out this important clarification.*

-Please describe what ansiotropic diffusion is
**Response:** *We thank the reviewer for the suggestion to clarify the meaning of anisotropic diffusion. While this concept was already mentioned in the Results section, we have now expanded the description slightly to improve clarity for readers less familiar with diffusion imaging. The revised text now explicitly defines anisotropic diffusion as the directional preference of water diffusion along white matter fibers, which underlies the biological interpretation of FA.*

-Please make sure to italicize gene names
**Response:** *We thank the reviewer for this formatting suggestion. In accordance with standard genomic nomenclature, we have now italicized all gene symbols throughout the manuscript and figure legends where appropriate.*

DISCUSSION
Major issues:
-Please discuss the gSEM model and whether the loadings make sense. Why is that insomnia loaded significantly less onto mvFibroPsych despite having a similar genetic correlation to fibromylagia, anxiety, and depression?
**Response:** *We thank the reviewer for this thoughtful and important observation. Indeed, the GenomicSEM factor loading reflects not only the pairwise genetic correlation between each trait and the others, but also how well genetic variance of each trait is captured by the latent common factor, after accounting for the full genetic covariance structure among all included traits.*

*In our model, although insomnia shows moderate genetic correlations with fibromyalgia (rg ≈ 0.57), depression (rg ≈ 0.58), and anxiety (rg ≈ 0.55), its factor loading (0.63) was somewhat lower than those of depression (0.94), anxiety (0.89), and fibromyalgia (0.74). This suggests that a larger portion of genetic variance of insomnia may be trait-specific, or shared with other factors not captured by the mvFibroPsych latent dimension.*

*One possible explanation is that insomnia has a more heterogeneous genetic architecture, encompassing both psychiatric and circadian/sleep regulation pathways, some of which are not strongly aligned with the shared pain–psychopathology factor modeled here. We have now added a brief interpretation of the loading pattern in the Results and Discussion sections to reflect this point. We appreciate the reviewer for prompting this clarification.*

-Please discuss the GWAS results in the context of previous fibromyalgia genetic studies – did you replicate any previous signals? What loci were new? It would be interesting to include a pathway analysis on just the new loci

**Response:** *We appreciate the interest of the reviewer in the relationship between our findings and prior fibromyalgia GWAS. We note that none significant loci was reported in the BioMe dataset for fibromyalgia and the two suggestive loci were reported in the FinnGen dataset (rs139332433 and rs56132815) with genome-wide significance marginally (P = 4.65×10⁻⁸ and 4.94×10⁻⁸, respectively). In our multivariate GWAS of mvFibroPsych, these variants did not replicate at the genome-wide level.*

*Importantly, the common factor model in GenomicSEM reflects shared genetic architecture across fibromyalgia, insomnia, depression, and anxiety. As such, loci that are primarily specific to fibromyalgia alone, especially if they do not exhibit strong cross-trait pleiotropy, may not emerge as significant in the multivariate GWAS. This does not contradict the increased power of GenomicSEM, but rather reflects its selectivity for shared liability.*

*In contrast, we identified 64 genome-wide significant loci that were not detected in any of the input GWASs, including fibromyalgia, highlighting the utility of the multivariate model in uncovering novel pleiotropic signals. We now discuss this explicitly in the revised Discussion to clarify as follows:*

“It is noteworthy that some fibromyalgia-specific loci, including previously reported borderline-significant hits in the FinnGen dataset (rs139332433 and rs56132815), did not replicate in our multivariate model. This likely reflects the fact that mvFibroPsych captures shared, rather than disorder-specific, genetic effects.”
