# Supplementary material for: Shared latent genetic liability across fibromyalgia and psychiatric traits: Novel insights from genomic structural equation modeling
Source: PLoS Genet. 2026 Jan 23;22(1):e1012034. doi: 10.1371/journal.pgen.1012034 (PMC12867332; doi:10.1371/journal.pgen.1012034)
Supplement: Attachment — Submitted filename: Point-to-Point Response.docx [file pgen.1012034.s022.docx]

**Point-to-Point Response**

Reviewer #1: I thank the authors for providing additional information regarding their submission as requested.

In regard to their response to my previous comments, I have remaining disagreements, in addition to new concerns upon examining the provided supplementary information, particularly supplementary table S4 and S5.

**Major comments:**
1) The Manhattan plot in figure 2C shows the smallest SNP p-value of the latent mvFibroPsych factor to be ~1e-30. However, in supplementary table S4 ‘Lead SNPs and Genomic Loci Associated with mvFibroPsych’, multiple SNPs have a p-value lower than 1e-200. There is an inconsistency in what is plotted in the main figure of figure 2C and what is reported in the supplementary tables S4 and S5.
Furthermore, supplementary table S5 contains theoretically impossible results, such as for SNP rs979343, which shows a mvFibroPsych Z score of -31.050, while the input Z score of anxiety, depression, insomnia, and fibromyalgia range from -0.3 to 0.35.
Similar incoherent results are reported for rs7067621, rs6783689, rs667255, rs6459268, rs6424719, rs61023573, rs59608961, rs4940514, rs17797882, and rs150011668.
In my estimation, these SNP effects reported in supplementary table S4 and S5 are unreliable and must be investigated further. Alternatively, the correct estimates must be reported again in supplementary table S4 and S5, and be resubmitted.
**Response:** *We sincerely thank the reviewer for the careful examination of our results and for pointing out the inconsistencies between the Manhattan plot in Figure 2C and the corresponding SNP-level statistics reported in Supplementary Tables S4 and S5. We have carefully investigated this issue in depth.*

*Upon re-examination, we confirmed that the input Z-scores for each individual trait (anxiety, depression, insomnia, and fibromyalgia) were correct. However, we found that during the GenomicSEM model fitting step, a subset of SNPs yielded unstable standard errors (SEs) when the analysis was executed in parallel mode, leading to spuriously extreme P-values (e.g., smaller than 1×10⁻²⁰⁰). Importantly, this instability was not caused by incorrect input summary statistics, but rather to numerical instability within the parallel computation process of the GenomicSEM package when handling large-scale GWAS data.*

*After updating GenomicSEM to its most recent version and re-running the analysis without parallelization, we obtained stable and consistent SNP-level estimates across repeated runs. We then rederived the mvFibroPsych GWAS summary statistics using this corrected approach. Based on the revised results, we identified 49 lead SNPs, among which 32 were novel. Supplementary Tables S4 and S5 have been fully updated with the corrected effect estimates and P-values, and Figure 2 has also been redrawn accordingly.*

*Furthermore, all downstream analyses — including FUMA annotation, LCV, neuroimaging association, and multi-omics analyses — have been re-performed using the updated summary statistics. Encouragingly, despite changes in the number of lead SNPs, the major findings and biological interpretations remain largely consistent with our initial version.*

*We sincerely apologize for not detecting this GenomicSEM-related computational issue earlier. We are deeply grateful to the reviewer for the sharp and constructive observation that helped us identify and correct this technical artifact, thereby improving the robustness and reproducibility of our study.*

2) In response to my concern that fibromyalgia cases amount to 2% of the total case number across the 4 included traits in the study, the authors argue that GenomicSEM is effective in boosting the genetic signal of less powered traits by utilizing the genetic covariance with other, well-powered traits. They refer to publications by Rosoff et al. 2023, Grotzinger et al. 2019, and Chen et al. 2023, to underline how this has been applied in previous studies.

To start, I do not see a mention of GenomicSEM in the Chen et al. 2023 publication “Genomic atlas of the plasma metabolome prioritizes metabolites implicated in human diseases”, so it is unclear to me what I am supposed to take away from this article.

I agree with the authors that conducting GenomicSEM on a set of traits with varying sample sizes and statistical power is possible, however, this study design has consequences for the final interpretation of the results. It is in this interpretation that I find the way the discussion section in the paper is currently lacking.

To illustrate my point, the Rosoff et al. paper combines GWASs of 5 aging-related traits (frailty, healthspan, parental lifespan, longevity, and phenotypic age acceleration) into one general aging related latent factor. All functional annotations associated with this latent factor is interpreted as generally relevant for aging-related processes.
In the current paper, GWAS of fibromyalgia, depression, anxiety and insomnia are combined into the latent factor mvFibroPsych and significant functional annotations are consistently contextualized as fibromyalgia AND psychiatric disorders. See examples: “[…]comprehensive understanding of the shared genetic underpinnings of fibromyalgia and its associated psychiatric disorders”(p.7 line 136), or “[…]neurobiological mechanisms that contribute to the comorbidity between fibromyalgia and psychiatric disorders” (p30, line 642).

Hence when the authors are interpreting the results associated with the latent factor mvFibroPsych, they refer to the observed factors that make up the latent factor instead of interpreting the latent factor itself. This seems to me to be a fallacy in interpreting the current study design, which leads to inaccurate reporting given the underlying evidence. The latent factor mvFibroPsych might as well be termed mvInternalising or mvNegativeAffect and the connection to chronic pain would already seem less obvious.
In the very least, I require that this features more prominently in their discussion of study limitations and that reporting of results are deemed putative unless confirmed in additional SNP-to-latent factor analyses.
**Response:** *We sincerely appreciate the reviewer’s thoughtful and detailed comments. We agree that the interpretation of the mvFibroPsych latent factor should be framed with greater conceptual precision.*

*First, we have removed the citation to Chen et al. (2023), as suggested. The methodological justification for our use of GenomicSEM now relies on Grotzinger et al. (2019) and Rosoff et al. (2023), which more appropriately illustrate this modeling strategy.*

*Second, we fully acknowledge the reviewer’s point that the mvFibroPsych factor represents a statistically derived latent construct capturing shared genetic variance across fibromyalgia, depression, anxiety, and insomnia. It should therefore not be interpreted as a direct genetic signal of fibromyalgia or psychiatric disorders per se, but rather as a broader dimension of shared liability underlying these interrelated phenotypes.*

*In response, we have substantially revised the Discussion section to (1) clarify the conceptual scope of the mvFibroPsych factor, (2) emphasize that all biological and functional annotations are putative and should be interpreted as reflecting this shared latent dimension, and (3) explicitly acknowledge this as a key limitation of the study. We have also adjusted the phrasing throughout the manuscript—for example, replacing expressions such as “fibromyalgia and its associated psychiatric disorders” with “the mvFibroPsych latent factor, representing shared genetic liability across fibromyalgia and psychiatric traits.”*

*We are grateful to the reviewer for this insightful clarification, which has helped us strengthen the conceptual and interpretative rigor of our work.*

3) While I appreciate the authors efforts in conducting alternative GenomicSEM models showing Fibromyalgia with each of the three psychiatric disorders separately, sufficient model fit showing shared genetic covariance is not the main point. The main point is the resulting SNP associations of these separate latent models and their subsequent association with functional annotations. I still am not convinced that the majority of the reported results are at all relevant to fibromyalgia. This is underlined by the fact that among the 122 reported lead SNPs in the mvFibroPsych latent factor, 81 SNPs do not show nominal significance (Z score range from 1.96 to -1.96) in the individual Fibromyalgia GWAS, as seen in Supplementary table S5. In addition, for many of the 122 genome-wide significant lead SNPs there are discordance in the effect direction among the individual traits, which makes interpretation less straight forward as some downstream associations seem protective for some traits while risk increasing for others.
**Response:** *We thank the reviewer for these valuable comments and for highlighting the importance of evaluating the SNP-level effects and their functional relevance.*

*Following the reviewer’s suggestion, we re-ran the mvFibroPsych model using the updated and more stable version of GenomicSEM, and systematically examined heterogeneity across the four input traits (fibromyalgia, anxiety, depression, and insomnia) using Cochran’s Q test. The results show that only one lead SNP (rs1245129) exhibited significant heterogeneity after Bonferroni correction (Q_P = 0.00003 < 0.05/49), whereas the remaining SNPs did not show statistically significant heterogeneity (Table S5, updated). Although several SNPs showed varying directions of effect across traits, their differences were not statistically significant, suggesting an overall pattern of genetic homogeneity. This indicates that the associations captured by the mvFibroPsych factor primarily reflect shared genetic liability rather than trait-specific or divergent effects.*

*We fully agree with the reviewer that not all genome-wide significant SNPs from the latent model are directly relevant to fibromyalgia per se. Indeed, by design, mvFibroPsych was constructed to identify the common genetic component underlying fibromyalgia and psychiatric traits, rather than fibromyalgia-specific variants. The absence of nominal significance for many SNPs in the individual fibromyalgia GWAS therefore aligns with the conceptual aim of this model — to reveal shared, rather than disorder-specific, genetic mechanisms.*

*In the revised Discussion, we have explicitly clarified this point and emphasized that the mvFibroPsych associations should be interpreted as putative shared genetic signals across fibromyalgia and affective dimensions, rather than as evidence of direct fibromyalgia-specific effects. We are grateful to the reviewer for encouraging this clarification, which strengthens the interpretative rigor of our study.*

**Minor comments:**
1) On page 20, line 410-413, the authors write “[…]gene-set analyses revealed that mvFibroPsych has an enrichment in the pathways involved in synaptic function, AMPA glutamate signaling, benzodiazepine receptor, and potassium ion transporters activities (Table S8).” Table S8 shows that these gene-sets, except GOCC_SYNAPTIC_MEMBRANE, are not significant after bonferroni correction and should not be reported.

**Response:** *We thank the reviewer for carefully noting this issue. We fully agree that, according to the revised Table S8, only the GOCC_SYNAPTIC_MEMBRANE pathway remains significant after Bonferroni correction, whereas the enrichments related to other pathways, did not survive the multiple-testing adjustment.*

*In response, we have revised the text to report only the Bonferroni-significant gene-set (GOCC_SYNAPTIC_MEMBRANE). The remaining pathways are now described in Table S8 as suggestive (uncorrected) findings for completeness, but they are no longer highlighted in the main text or abstract.*

***We would like to express our deepest gratitude to the reviewer for the insightful and constructive feedback. The comments have been invaluable in improving the robustness, transparency, and scientific soundness of our study. We have carefully addressed all the concerns raised and made substantial revisions accordingly. We sincerely hope that these in-depth modifications will meet the reviewer’ expectations and further enhance the quality of our manuscript. We truly appreciate the reviewers’ time, patience, and thoughtful engagement with our work.***
