# Supplementary material for: Shared latent genetic liability across fibromyalgia and psychiatric traits: Novel insights from genomic structural equation modeling
Source: PLoS Genet. 2026 Jan 23;22(1):e1012034. doi: 10.1371/journal.pgen.1012034 (PMC12867332; doi:10.1371/journal.pgen.1012034)
Supplement: Attachment — Submitted filename: Response_to_Reviewers_auresp_3.docx [file pgen.1012034.s023.docx]

**Point-by-Point Response**

Reviewer #1: I much appreciate the changes made to this version of the paper. Thank you to the authors for thoroughly repeating their work to increase the robustness and reliability of the work they present. I particularly think that the discussion has improved and presents sufficient nuance and statistical caution with regards to the interpretation of the results. I only have two remaining minor comments, after which I would regard this paper ready to be suitable for publication.

**Response:** *We sincerely thank the reviewer for the positive and encouraging comments on the revised manuscript. We greatly appreciate the recognition of our efforts to improve the robustness, reliability, and interpretative rigor of this work. We are also grateful for the reviewer’s constructive suggestions and have carefully addressed the remaining minor comments accordingly.*

1) The authors have thoroughly rephrased interpretation of results from their study design, replacing formulations like "significant for fibromyalgia and its associated psychiatric disorders” with “significant for the mvFibroPsych latent factor, representing shared genetic liability across fibromyalgia and psychiatric traits.”. This is good, but I would argue that the title of the study ("Uncovering the Psychiatric Underpinnings of Fibromyalgia:[...]") still assumes that presented findings are all associated with fibromyaligia and the other psychiatric disorders. I would suggest to change the title to avoid this assumption.
**Response:** *We thank the reviewer for this important observation. We fully agree that our original title could still be interpreted as implying fibromyalgia-specific psychiatric mechanisms, which would not be fully consistent with our revised interpretation of the mvFibroPsych latent construct as reflecting a shared genetic liability across fibromyalgia and internalising psychiatric traits. To ensure full conceptual consistency between the study design, interpretation, and presentation, we have therefore revised the title to:*

“Shared Latent Genetic Liability Across Fibromyalgia and Psychiatric Traits: Novel Insights from Genomic Structural Equation Modeling.”

*We believe that this revised title more accurately reflects the latent factor framework adopted in this study, avoids over-attribution of the findings to fibromyalgia per se, and aligns with the conservative scope of inference now emphasized throughout the manuscript.*

2) Looking at the Manhattan plot in figure 2C: the top SNP (rs4865477, p-value = 2.31e-24) is highly suspicious. Suppl Table S6 shows other SNPs in >0.6 LD with this SNP having p values of ~0.045. Additionally in Supple Table S5, the input Z-scores of the 4 traits for rs4865477 are all between -1.96 and 1.96, ie. not nominally significant, yet somehow combining these input z-scores produces the most significant association with the latent factor.
Though it is a bit difficult see from figure 2C, several of the other novel hits (on chr 23, chr 9 and chr 8 appear to be significant without any surrounding SNPs showing signs of significance as well. These singular significant SNPs should be carefully investigated or removed outright from results and downstream analysis, as they are biologically implausible.

**Response:** *We thank the reviewer for this thoughtful comment. We carefully re-examined the loci highlighted in the comment, including rs4865477. Multiple members of our research team independently re-ran all GenomicSEM analyses using separate computational environments and software installations, and identical results were obtained, excluding implementation or data-processing errors.*

*We acknowledge that rs4865477 shows modest marginal Z-scores in each individual univariate GWAS and does not display classical clusters of surrounding SNPs with strong associations in the mvFibroPsych Manhattan plots. However, both observations are expected properties of latent common factor GWAS using GenomicSEM and do not indicate spurious associations.*

*First, GenomicSEM does not rely on marginal single-trait significance but estimates SNP effects on a latent liability dimension by weighting marginal effects by the genetic covariance structure among traits. Consequently, variants exhibiting small but directionally consistent pleiotropic effects across genetically correlated traits can yield extremely small P-values in latent common factor GWAS, even when no individual marginal effect reaches nominal significance. This phenomenon was explicitly reported in the original GenomicSEM application, which identified 27 genome-wide significant loci for a general psychopathology latent factor that were not detected in any contributing univariate GWAS (Grotzinger et al., Nature Human Behaviour, 2019).*

*Second, the absence of classical LD-supported clusters (“surrounding SNPs”) is also an anticipated feature of latent pleiotropic loci. Latent common factor GWAS tests whether a SNP projects stably onto a multivariate covariance-defined liability dimension rather than testing association with a single phenotype. Nearby SNPs in LD may not share the same multivariate pleiotropic configuration and therefore may not exhibit comparable latent associations, resulting in isolated lead variants without dense LD clouds in the latent common factor Manhattan plots.*

*Importantly, rs4865477 did not show significant Q_SNP, indicating that its effect is well captured by the common-factor model and reflects shared genetic liability rather than trait-specific heterogeneity. Only one locus (rs1245129) in our study exhibited significant Q_SNP after multiple-testing correction, and this locus is explicitly interpreted with caution.*

*Taken together, the modest marginal Z-scores, absence of classical surrounding SNP clusters, and significant P-values observed for rs4865477 and other lead SNPs are consistent with the theoretical and empirical properties of GenomicSEM-based latent common factor GWAS. Excluding such loci solely based on these characteristics would therefore be methodologically inappropriate and would risk removing precisely those pleiotropic variants that multivariate GWAS is designed to detect. We have added clarifying text to the Methods, Results, and Discussion sections to explicitly reflect this interpretation framework.*

*We thank the reviewer again for this insightful and constructive comment, which has helped us to further clarify the methodological rationale and interpretation framework of our latent factor GWAS. We believe that the revisions prompted by this comment have substantially strengthened the transparency and rigor of the manuscript.*

***Reference:***

*- [Grotzinger](https://pubmed.ncbi.nlm.nih.gov/?term=Grotzinger+AD&cauthor_id=30962613) et al. (2019) Genomic structural equation modelling provides insights into the multivariate genetic architecture of complex traits. Nat Hum Behav. 2019 May;3(5):513-525.*
